# Pathology of General Practice Curriculum from the Viewpoint of Students and Professors

**DOI:** 10.30476/jamp.2021.87035.1287

**Published:** 2021-04

**Authors:** SHAPOUR BADIEE AVAL, ALI EMADZADEH, MARYAM POURSHIRAZI, HOSEIN KARIMI MOONAGHI, SEYED JAVAD HOSSEINI, ZAHRA ABBASI SHAYE, GOLNAZ SABOURI, FATEMEH HOSSEINI DOLATABADI

**Affiliations:** 1 Department of Complementary and Chinese Medicine, School of Persian and Complementary Medicine, Mashhad University of Medical Sciences, Mashhad, Iran; 2 Department of Medical Education, Faculty of Medicine, Mashhad University of Medical Sciences, Mashhad, Iran; 3 Clinical Research and Development Center, Deputy of Treatment, Mashhad University of Medical Sciences, Mashhad, Iran; 4 Department of Medical Biotechnology, School of Medicine, Mashhad University of Medical Sciences, Mashhad, Iran; 5 Clinical Research and Development Center, Faculty of Medicine, Mashhad University of Medical Sciences, Mashhad, Iran; 6 Department of Educational Management, School of Medicine, Mashhad University of Medical Sciences, Mashhad, Iran

**Keywords:** Curriculum, Pathology, General practitioners, Students, Medical schools

## Abstract

**Introduction:**

Based on the needs of the health system, continuous revising and monitoring are essential for General Practice (GP) Curriculum. The present study was conducted to investigate the diseases of GP Curriculum based on the students’ and professors’ viewpoints.

**Methods:**

This cross-sectional study was carried out at Mashhad University of Medical Sciences in 2018. A total of 80 GP students of internship and 71 professors of the faculty of medicine in clinical and basic science disciplines were enrolled in the study using quota and convenience sampling methods, respectively. Two self-made, reliable, and validated 4-point scale questionnaires (ranging from totally agree to totally disagree) were used to collect the data on the viewpoints of students and professors on the diseases of GP curriculum. The mean score and percentage of agreement between professors and students on the incidence of each disease were calculated.

**Results:**

The highest and lowest rates of agreement between the professors and students in terms of the presence of curriculum diseases belonged to the echolalia curriculum with a mean and standard deviation of 1.92±0.68 and the dean denial with a mean and standard deviation of 2.0±6.68, respectively. The results showed a statistically significant difference between the viewpoints of professors and students regarding the diseases of the carcinoma of the curriculum (P<0.001), idiopathic colitis (P<0.001), the schizophrenia of the curriculum (P=0.01), and echolalia curriculum (P=0.01).

**Conclusion:**

The present study showed that professors and students were all in agreement about 12 out of 13 diseases of the GP curriculum. Thus, educational planners in Iran’s medical schools should focus on the continuous evaluation and the necessity of curriculum revision, as one of the priorities of the educational system.

## Introduction

Today, there are concerns about learning in the medical education system. An effective curriculum determines the success of learning ( 1 ). The curriculum is defined as the activities offered to students by the faculty and is dynamic, rather than stationary. In fact, it is a product of planning and implementing. Dr. Abrahamson, a consultant on education and curriculum issues at the American Medical Schools, identified 13 curriculum diseases during his visit to the medical school (Table 1) ( 2 - 4 ). One of the most essential duties of an educational system is providing the health system with a comprehensive written curriculum for training human resources, including general practitioners. It should respond to the changing needs of the community and assist the health system in fulfilling its tasks by training skilled and competent human resources as well ( 5 ). Over the past century, many developments have occurred in the medical education system worldwide that caused Iran’s medical education to be far behind. The efforts of Abraham Flexner, from the 1910s to the 1940s decades, led to the first wave of reforms in the medical education system of the Western world. This reformation resulted in a systematic approach to medical education and an emphasis on the importance of educational management and leadership for training physicians. The second wave of reforms mainly manifested itself as changes in educational methods and positions. The third wave of reforms took place from 1990 to 2000. It was based on The Edinburgh Declaration, which emphasized the effect of medical education on providing better health services. To achieve this goal, it was recommended to use active training methods and integrate basic knowledge and clinical skills ( 6 , 7 ). With the increasing and variable needs of the country as well as the development of knowledge and technology along with innovation and reform practices in medical education around the world, there is a need for our country’s curriculum to be adapted to these changes. An overview of the revision phenomenon in the country shows that there has been no comprehensive and thorough revision of the structure and method of education. Moreover, the educational indicators of the world's top medical schools such as systematic education, early clinical exposure, and horizontal and vertical integration have been fulfilled in 9.1% of the curriculum of medical schools in Iran ( 5 ).

**Table 1:** Definitions for diseases of curriculum according to Dr. Abrahamson (2)

Disease	Definition
Curriculosclerosis	Hardening of the categories.
Carcinoma of the Curriculum	Uncontrollable growth of one segment or component of the curriculum.
Curriculoarthritis	A condition affecting the articulations between adjacent or related segments of the curriculum and may affect horizontal or vertical articulations; that is, one can find this disease affecting the relationship between one subject taught in the first year and another in the second. Or one can find evidence of this disease in the relationship between the two subjects taught simultaneously.
Curriculum Dysesthesia	The curriculum appears to be in good health and yet a feeling that something is not quite right persists.
Iatrogenic Curriculitis	The curriculum is subject to too much tampering or meddling that there is no opportunity for a thoughtful review.
Curriculum Hypertrophy	As each frontier of knowledge is pushed back, each discipline tends to want to include the discoveries in the curriculum.
Idiopathic Curriculitis	It is the teaching that is bad, not the curriculum.
Intercurrent Curriculitis	A reflection of the incompatibility or unresponsiveness of the curriculum to concurrent social problems.
Curriculum Ossification	The curriculum appears as if “cast in concrete”.
Curriculum Schizophrenia	Dispersion and lack of consistency and coordination between basic science and clinical course.
Interventional Curriculitis	It is due to the lack of attention on current social issues by the curriculum. Although the society is in demand for public issues, it affects colleges in which scientific themes are emphasized.
Dean Denial Curriculum	Dean’s inability to recognize the curriculum problems.
Hereditary Curriculitis	The new dean inherits a new curriculum problem.
Echolalia Curriculum	Echolalia is the repetition or echoing of verbal utterances made by students in response to the excessive content of the curriculum.

Studies show that the GP alumni do not consider their skills and qualifications desirable in self-evaluations. Although there is controversy among medical education practitioners and experts, evidenced-based data show that the majority of the experts are relatively satisfied with the educational approach in GP curriculum. Others express different problems in the education process ( 6 ).

 One of the most important and effective ways of foreseeing and revising a curriculum is to collect feedback from those who have experienced it. Feedbacks determine the success of the curriculum and its areas of improvement. The most important resources for providing feedback on educational activities are professors, alumni, and students. Many studies have analyzed this feedback through a variety of methods ( 8 - 11 ). Since every curriculum is designed to train the skilled and specialized human resources, continuously revising and monitoring of the curriculum in academic courses and offering a comprehensive and developed curriculum based on the needs of the health system are necessary for all medical and health professions. Therefore, the present study was conducted to investigate the pathology of the General Practice curriculum based on the students' and professors' viewpoints.

## Methods

80 GP students of internship and 71 professors of the faculty of medicine in clinical and basic science disciplines were enrolled in the study using quota and convenience sampling methods.

In this cross-sectional study, GP curriculum pathology questionnaire was distributed among 100 GP students of internship of Mashhad University of Medical Sciences and 100 professors (20 basic science professors and 80 clinical professors) of this university. Of them, 80 students and 71 professors completed the questionnaire. The students were selected via convenient sampling and according to the inclusion criteria. Professors were selected based on the sample size using quota sampling. That is to say, the participants enrolled the study in accordance with the percentage of faculty members in Basic Sciences and Clinical disciplines using convenient sampling.

The total number of faculty members of Mashhad University of Medical Sciences was 600: 480 clinical science professors, 80 of whom were selected, and 120 basic science professors, 20 of whom were enrolled in the study. 

Inclusion criteria were as follows:

Intern students of Mashhad University of Medical Sciences who had completed all courses of general medicine (basic science, physiopathology, and externship) at this University and were satisfied with the study
Faculty members of basic and clinical sciences who had teaching experience in general medicine, had at least one semester of experience, and were willing to participate in the study.

In order to collect the data, we used two researcher-made questionnaires. The first questionnaire, designed to evaluate the students’ viewpoints on the diseases of GP curriculum, consisted of 8 demographic questions and 19 questions about the diseases in the curriculum. The second questionnaire was about the professors' viewpoints and included 4 demographic questions and 17 questions about the diseases of GP curriculum. These two researcher-made questionnaires were designed based on Dr. Abrahamson's (2 and 3) curriculum diseases and their validity and reliability were measured. The scoring method in both questionnaires (except for demographic questions) was a 4-point Likert Scale (strongly agree, agree, disagree, and strongly disagree). Scores one and four were given to “strongly agree” and “strongly disagree”, respectively. Each questionnaire contained some questions with the semantic concept and scoring in reverse order. Scores were reversed while describing and analyzing the questions. According to the experts’ consensus (including two medical education and two community medicine specialists), a score below or equal to 2.5 was considered as the presence of the disease. Qualitative and quantitative methods were used to determine the content validity of the questionnaires. To assess the qualitative validity of the questionnaires, we asked 10 medical and social education experts to correct the questions in terms of grammar, wording, and phrasing. The content validity was studied quantitatively using Content Validity Ratio (CVR) and Content Validity Index (CVI). To assess the content validity of the questionnaires, the experts were asked to evaluate the questions according to a three-section range, i.e. essential, beneficial, and unessential. The CVR was then calculated based on the given responses. It should be noted that the acceptable range depends on the number of experts. In the present study, the acceptable range was considered 0.62 by the judgment of 10 experts. In other words, if the calculated CVR for each item is above or equal to 0.62, the content validity of the item will be confirmed. In our study, the content validity for both questionnaires was above 0.6. In addition, CVI was determined using the average CVR for the rest of the items which was confirmed by the figure above or equal to 0.8 for both questionnaires. 

The internal consistency coefficient (Cronbach's alpha) was used to calculate the internal reliability of the questionnaire, which in our study was 0.73 and 0.78 in the students and professors' questionnaires, respectively. The printed questionnaires were distributed among the participants by two trained interviewers. The students' questionnaires were distributed among the interns of Mashhad University of Medical Sciences in Imam Reza and Ghaem hospitals. The professors' questionnaires were also distributed among the professors of basic sciences and clinical science courses at Mashhad University of Medical Sciences in Imam Reza and Ghaem hospitals. The participants were informed about the objectives of the study and informed consent was obtained. The questionnaires were anonymized and the participants were assured that their information would be kept confidential.

### Statistical analysis

After the questionnaires were completed, data analysis was carried out using SPSS statistical software version 22. The descriptive statistics including mean and frequency were used to describe the sample characteristics. The relationships between qualitative variables were analyzed using chi-square test and those between quantitative variables were studied using t-test one-way ANOVA. The correlations between quantitative variables were studied using Pearson correlation test. P values less than 0.05 were regarded as statistically significant. 

## Results

In the present study, 80 students and 71 professors completed the questionnaires. The mean age of the students was 25.24±1 years old (minimum=22 and maximum=35). The mean number of major courses passed by students was 2.44±0.84 (minimum=2 and maximum=4). The mean number of minor courses passed by students was 4.2±2.77 (minimum=0 and maximum=11). The mean age of the professors was 45.37±8.14 years old (minimum=34 and maximum=68). The mean years of teaching experience for the professors was 9.62±6 (minimum=1 and maximum=35). 

The results showed that the highest and lowest rates of agreement among the students belonged to the echolalia (82.50%) and the ossification of the curriculum (31.30%) in the clinical course, respectively. It was also found that curriculum carcinoma had the highest rate (88.80%) and the schizophrenic curriculum had the lowest rate of agreement (34.1%) in basic sciences course among the students. 

Having analyzed the scoring of the diseases in the curriculum, we considered the scores below or equal to 2.50 as the approval of the presence of the disease in the educational curriculum, and scores higher than 2.50 were considered as the disapproval of the presence of the disease in the curriculum. According to the viewpoints of all professors (clinical and basic), the highest rate of agreement among them about the diseases in the curriculum belonged to the echolalia curriculum and interventional curriculum with the mean and standard deviation of 2.06 ± 0.61 (Table 2). The agreement was defined as the sum score of responses to “strongly agree” and “agree” items and the disagreement was calculated by adding the scores of responses to “strongly disagree” and “disagree” items.

**Table 2:** The average rate of agreement on the scores of the diseases in the curriculum from professors' viewpoints

Variable (Disease)	Mean±SD	Min	Max
Carcinoma of the Curriculum	2.38±0.84	1	4
Curriculosclerosis	2.27±0.78	1	4
Curriculum Dysesthesia	2.19±0.78	1	4
Iatrogenic Curriculitis	2.18±0.77	1	4
Curriculum Hypertrophy	2.40±0.64	2	4
Idiopathic Curriculitis	2.54±0.58	1	4
Interventional Curriculitis	2.06±0.60	1	4
Curriculum Ossification	2.36±0.57	1	3
Curriculum Schizophrenia	2.51±0.53	1	3
Hereditary Curriculitis	2.37±0.67	1	4
Dean Denial Curriculum	2.59±0.65	1	4
Echolalia Curriculum	2.06±0.61	1	4
Curriculoarthritis	2.27±0.39	1.25	3.25

According to the students' viewpoints, the echolalia curriculum with the mean and standard deviation of 78.55±1.0 rated for the highest agreement followed by *interventional curriculum* with the mean and standard deviation of 1.98±0.98. From the students' point of view, only ossification disease with a mean and standard deviation of 2.62±0.58 was not observed in the curriculum (Table 3).

**Table 3:** The average rate of agreement on the scores of the diseases in the curriculum from students' point of view

Variable (Disease)	Mean±SD	Min	Max
Carcinoma of the Curriculum	2.01±0.61	1	3.5
Curriculosclerosis	2.01±0.61	1	3.5
Curriculum Dysesthesia	2.01±0.61	1	3.5
Iatrogenic Curriculitis	2.24±0.61	1	4
Curriculum Hypertrophy	2.45±0.58	1	4
Idiopathic Curriculitis	2.08±0.63	1	4
Interventional Curriculitis	1.98±0.98	1	4
Curriculum Ossification	2.62±0.70	1	4
Curriculum Schizophrenia	2.26±0.96	1	4
Echolalia Curriculum	1.78±0.55	1	3
Curriculoarthritis	2.32±0.59	1	3.5

According to the viewpoints of the participants (professors and students), the highest rate of agreement was observed for the echolalia curriculum with the mean and standard deviation of 1.92±0.68. Moreover, the dean denial curriculum with the mean and standard deviation of 2.6±0.68 showed the lowest agreement on the presence of diseases in the curriculum (Table 4).

**Table 4:** The average rate of agreement on the scores of diseases in the curriculum from the students and professors' point of view

Variable (Disease)	Mean±SD	Min	Max
Carcinoma of the Curriculum	2.19±0.81	1	4
Curriculosclerosis	2.14±0.75	1	4
Curriculum Dysesthesia	2.10±0.82	1	4
Iatrogenic Curriculitis	2.22±0.71	1	4
Curriculum Hypertrophy	2.43±0.66	1	4
Idiopathic Curriculitis	2.31±0.73	1	4
Interventional Curriculitis	1.98±0.80	1	4
Curriculum Ossification	2.49±0.77	1	4
Curriculum Schizophrenia	2.25±0.82	1	4
Hereditary Curriculitis	2.37±0.68	1	4
Dean denial Curriculum	2.60±0.68	1	4
Echolalia Curriculum	1.92±0.68	1	4
Curriculoarthritis	2.29±0.62	1	3.75

After the relationship between the students' viewpoints on each disease and their characteristics as secondary objectives were investigated, the ANOVA test only showed a significant statistical difference between the year of entry and the scores of students' viewpoints on the carcinoma curriculum (p=0.04) and echolalia curriculum (p=0.001). The post-hoc test showed that the score of students' comments about the carcinoma curriculum in 2012 was significantly higher than that of 2010. The same result was found for the score of students' comments about the echolalia curriculum in 2013 as opposed to that of 2010, 2011, and 2012. No significant relationship was observed between the students' viewpoints on each disease and other individual characteristics.

The relationship between the professors' opinions about each disease and their characteristics was studied using Pearson correlation test, which showed no significant relationship between the professors' academic rank and their viewpoints on the studied diseases (p>0.05). There was a significant correlation between the professors’ years of teaching experience and their viewpoints on the echolalia curriculum.

Moreover, according to the relationship between the students and professors' viewpoints on each disease, ANOVA test showed a significant difference between the scores of Carcinoma of the Curriculum (P<0.001), Idiopathic Curriculitis (P<0.001), Curriculum Schizophrenia (P=0.01) and Echolalia Curriculum (P=0.01). The post-hoc test showed that the differences were mostly related to the students' opinions about clinical professors.

The t-test showed that except for the viewpoints on Curriculum Hypertrophy that female students scored 2.55±0.55 and male students scored 2.84±0.60, no difference was observed between the two genders regarding other viewpoints. Also, independent t-test showed no significant difference between the scores of the diseases of the curriculum from male and female professors' points of view (p=0.064).

The correlation between the participants' viewpoints for each disease and their viewpoints on other diseases was obtained through Pearson correlation test. The test showed a significant moderate correlation between the professors and students' opinions about carcinoma in the curriculum and their viewpoints on Hypertrophic Curriculum (p<0.001, r=0.42) and Echolalia Curriculum (p<0.001, r=0.5). There was a poor correlation between their perspectives on other diseases of the curriculum. In some cases, there was no significant correlation.

## Discussion

Given the fact that the field of medicine is progressing rapidly and also educational methods are being upgraded, reviewing and updating the curriculum of a faculty is of great importance. Thus, there is a need to explore how necessary changes may be discovered to improve the curriculum. One of the most important ways to get feedback on the curriculum in educational planning is to evaluate the opinions of professors and students in a certain discipline. It should be noted that no similar study has been carried out so far that has examined the pathology and diseases of the general practice curriculum. However, to the extent possible, the results obtained from this study are compared to those of other studies that have investigated the GP curriculum. Based on the scores obtained from the professors' comments (Basic and Clinical Science courses), Echolalia Curriculum, Interventional Curriculitis, Iatrogenic Curriculitis, Curriculum Dysesthesia, Curriculosclerosis, Curriculoarthritis, Hereditary Curriculitis, Curriculum Ossification, Carcinoma in the Curriculum, and Curriculum Hypertrophy ranked from the highest to lowest scores in GP Curriculum, respectively. From the students’ point of view, only the Curriculum Ossification was not observable in the GP Curriculum. In general, students and professors reached agreement on 12 diseases out of 13 studied diseases of the GP curriculum. Echolalia Curriculum ranked for the highest score of agreement among the participants and the lowest agreement score belonged to the Dean Denial Curriculum. According to the correlation between the participants' viewpoints in terms of each disease and their viewpoints on other diseases analyzed by Pearson correlation test, the results showed that a moderate and significant correlation was found between the viewpoints of the participants on Carcinoma in the Curriculum and their viewpoints on Curriculum Hypertrophy and Echolalia Curriculum. There was a poor correlation between their perspectives on other Diseases of the Curriculum. In some cases, there was no significant correlation.

One of the diseases in the curriculum that was agreed by most students and professors in this study was Curriculoarthritis with the mean agreement of 29.62±2.0. This disease is associated with a lack of horizontal and vertical articulations in the curriculum. Amiri et al. studied horizontal and vertical integration in the GP curriculum in Hamadan, in which 69.35% of the participants believed that the horizontal integration program consisted of appropriate contents and 19.79% of all agreed on the vertical integration program. 50% of the professors also agreed to implement the current vertical integration program. Thus, according to their study, more than half of the students and professors were satisfied with the reform ( 8 ). Moreover, in another study at Shiraz University of Medical Sciences, more than two-thirds of the students believed that the integration was an encouragement for more participation in the course, and overall, the students evaluated both horizontal and vertical integrations as positive ( 9 ). Another study on the quality of GP Curriculum showed that the quality of education was almost weak based on the Likert scale. In terms of teaching based on the integration of basic and clinical courses, it was also reported that the mean score was low, indicating the need for revision of the implementation of the integration program at the university ( 12 ).

In the present study, Curriculum Schizophrenia was another disease in the GP Curriculum agreed by professors and students with the mean agreement of 25.82±2.0. This disease refers to a lack of consistency and coordination between basic sciences and clinical course. In a study by Chun et al. in Taiwan, 21% of the students believed that there was no proper relationship between basic sciences and clinical course ( 12 ). However, in the AAMC (American Medical Schools Association) report, only 8.5% of the students believed that there was no proper relationship between basic sciences and clinical course. This is not far-fetched based on the American medical education system because it is 35 or more years that the graduates' feedbacks are given to colleges annually and necessary changes are made ( 13 ). This reminds curriculum revision at Mashhad University of Medical Sciences.

Carcinoma in the curriculum with the mean agreement rate of 19.2±0.81 was another disease agreed by the professors and students in this study that was related to the growth of the contents of the curriculum. A study conducted in Shiraz, similar to the present study, showed the participating professors’ dissatisfaction in terms of the volume of the course content ( 9 ).

Curriculum Dysesthesia refers to a curriculum that seems to be in good health but has problems and errors in implementation and does not focus on the main goals. In a study by Jalalvandi et al., only half of the students expressed a positive viewpoint for areas of educational goals and curricula ( 14 ).

Idiopathic Curriculitis and Iatrogenic Curriculitis were agreed by professors and students in this study. Idiopathic Curriculitis indicates that the curriculum is good and appropriate, but the provided training and teaching are insufficient and unacceptable. The iatrogenic curriculum is subject to constant tampering or meddling. In a study by Jalili et al., 28.4% of the participants were satisfied with the quality of the medical education, and only 27% agreed that they had the necessary skills to work as a general practitioner ( 15 ). Mostafavian et al. examined the perspectives of senior medical students at Islamic Azad University of Mashhad and reported that 46% of participants were satisfied with the quality of medical education and 50% of them believed that they did not have the skills required for working as a general practitioner ( 16 ). A research carried out in Taiwan showed that 70.7% of the students were satisfied with the quality of medical education ( 17 ). Moreover, in the AAMC (American Medical Schools Association) report in 2018, 91.8% of the students were satisfied with the quality of education ( 18 ).

In the current study, a statistically significant difference was observed between the mean scores of the professors and students' viewpoints on diseases in the curriculum, namely Carcinoma in the Curriculum, Idiopathic Curriculitis, Curriculum Schizophrenia, Dean Denial Curriculum, and Echolalia Curriculum. A study conducted by Baqiyatallah University of Medical Sciences evaluated the quality of the GP Curriculum according to the national and international standards. The data were collected from two sample groups consisting of students and professors of Baqiyatallah University of Medical Sciences in basic sciences and clinical courses. The results of the study were similar to those of the present study. A statistically significant difference was found between the professors and students' viewpoints. The difference can be attributed to the differences in the collected data and in the subjects’ participation in the process of developing the university curriculum ( 19 ). 

### Strength and Limitation

To date, a few studies have investigated the views of stakeholders on the pathology of the general practice curriculum and most of them have examined limited diseases from the perspective of only professors or students. This is the first study in Iran which has thoroughly and comprehensively examined all curriculum diseases from the perspective of both professors and students who are the main target population for better implementation of the general practice curriculum.

The limitation of the present study was the lack of cooperation and responsiveness among the professors and students, especially clinical professors in completing the questionnaire.

## Conclusion

Our study showed that professors and students agreed with 12 out of 13diseases of GP curriculum. Echolalia Curriculum ranked for the highest mean score of agreement between professors and students. The significant moderate correlation between Carcinoma in the Curriculum and Echolalia Curriculum indicates excessive content of the GP Curriculum that leads to the repetition, or echoing of verbal utterances made by students. Furthermore, it should be noted that the significant correlation between Carcinoma in the Curriculum and Curriculum Hypertrophy shows that the new training items were added to the curriculum without deleting the previous ones. This led to the growth of the component of the curriculum and consequently increased the rate of Echolalia Curriculum. Accordingly, given the fact that the main objective of universities and training centers is to provide skilled human resources required for the society and that the field of medicine is progressing rapidly and education methods are being upgraded, reviewing and updating the curriculum of a faculty is of great importance. Thus, there is a need to explore how necessary changes may be discovered to improve the curriculum and more focus should be made on the curriculum and quality of medical education. The results of this study can be used for reviewing of GP Curriculum and diagnosing correct continuous evaluation, providing constructive feedback to students and professors. Continuous supervision on teaching following GP Curriculum requires revision and periodic evaluations of the curriculum. Therefore, serious measures are required to be taken by officials to rigorously review and resolve the defects of the medical curriculum. The results of this study can be used in the review of general medical education curriculum.
